# Real-world evidence on second line chemotherapy in advanced biliary tract carcinoma refractory to gemcitabine and oxaliplatin (GemOx)

**DOI:** 10.1186/s12885-026-16009-y

**Published:** 2026-04-17

**Authors:** Hanna Lagenfelt, Hakon Blomstrand, Emma Gränsmark, Nils O Elander

**Affiliations:** 1 Department of Biomedical and Clinical Sciences, Linköping University, Linköping, Sweden; 2 Department of Clinical Chemistry, Region Östergötland, Linköping, Sweden; 3 Department of Clinical Pathology, Region Östergötland, Linköping, Sweden; 4 Clinical Department of Oncology in Kalmar, Region Kalmar, Kalmar, Sweden; 5 Clinical Department of Oncology in Linköping, Linköping, Region Östergötland Sweden

**Keywords:** Biliary tract neoplasm, Chemotherapy, Second line treatment, Gemcitabine, Oxaliplatin, Palliative care, Real-world evidence, Capecitabine

## Abstract

**Background:**

Advanced biliary tract carcinoma (aBTC) comes with poor prognosis. Gemcitabine with oxaliplatin (GemOx) has emerged as a pragmatic alternative to gemcitabine with cisplatin and has been a preferred first line palliative option at many centres around the world. The evidence on second line chemotherapy following treatment failure of GemOx is scarce.

**Methods:**

A retrospective multicentre cohort study in the Southeast healthcare region of Sweden, covering nine years (2011–2020) and three hospitals, was conducted. All patients with aBTC who had received first line GemOx and any second line treatment thereafter were included. Treatment patterns and clinicopathological prognostic parameters were explored.

**Results:**

In a cohort of 121 patients with aBTC who had received GemOx in the first line setting, 57 (47%) had proceeded to any type of second line systemic therapy and were included in this study. The most used regimen was capecitabine single agent in 37 (64.9%) patients followed by intravenous 5-FU (9, 15.8%). Median overall survival (OS) in the total cohort was 4.1 months (95% CI 3.2-5.0). Six- and twelve-month survival rates were 35.6% and 18.6%. Median time to treatment failure (TTF) was 1.8 months (95% CI 1.5–2.2). Performance status (PS) 2, according to Eastern Cooperative Oncology Group (ECOG), was an independent predictor for poor OS and TTF. Progression free survival (PFS) of < 6 months during first line treatment and extrahepatic localisation were independent predictors for poor OS. A nomogram was established using ECOG PS, 1st line PFS and primary tumour site. An exploratory prognostic model showed that patients with high total score in the nomogram had OS of 2.9 months compared to 4.3 months in patients with intermediate score and 13.1 months in patients with low score.

**Conclusion:**

Patients with aBTC receiving second line chemotherapy following first line GemOx have very dismal prognosis. PS 0–1, first line PFS ≥ 6 months and intrahepatic disease localisation indicate better prognosis. These data highlight the need for improved and personalised treatment strategies in refractory aBTC.

## Background

Biliary tract carcinomas (BTC), including intrahepatic cholangiocarcinoma (iCCA), perihilar cholangiocarcinoma (pCCA), distal cholangiocarcinoma (dCCA) and gallbladder carcinoma (GBC), are a rare group of malignancies with poor prognosis [1, 2]. Many patients present with locally advanced or metastatic disease precluding curative intent resection, whereas others are detected early, at resectable stages yet being at high risk of relapse even if radical surgery has been performed. For patients with advanced BTC (aBTC), palliative systemic therapy remains the only way of potentially controlling the disease and extend life expectancy.

Over the past 15 years, gemcitabine with cisplatin has been the gold standard upfront treatment in patients with aBTC [3], in recent years with the optional addition of an immune checkpoint inhibitor [4, 5]. As an alternative to GemCis, many centres have utilized gemcitabine plus oxaliplatin as a pragmatic and well tolerated regimen, with outcomes comparable to what has been reported for GemCis [6–9]. Unfortunately, duration of response to gemcitabine-based regimens is usually limited, and a majority of patients experience primary or acquired resistance to the therapy. In a subset of iCCA refractory to chemotherapy, tailored treatment with drugs targeting actionable molecular alterations in genes such as FGFR2 and IDH has emerged as a novel therapeutic approach [10, 11], but for the vast majority of patients with aBTC best supportive care with or without a second line of conventional chemotherapy remain the only viable options.

The evidence on second line conventional chemotherapy in patients with aBTC progressing on first line gemcitabine-based therapy is limited. In patients who received upfront GemCis, the ABC-06 trial revealed a minimal benefit from FOLFOX vs. best supportive care (median OS 6.2 vs. 5.3 months) [12]. For patients who received first line GemOx, and who have no targetable genetic alterations or access to novel drugs, even less is known about potential benefit from further lines of conventional chemotherapy.

The present study aimed to explore treatment patterns and outcomes of second line chemotherapy in a real-world cohort of all patients with aBTC and who had progressed on first line GemOx chemotherapy. In addition, potential prognostic parameters were analysed, to identify groups of patients with particularly short or long survival.

## Methods

### Patients

A retrospective multicentre cohort study was conducted in the Southeast healthcare region of Sweden, covering approximately 1.1 million citizens across the oncology departments of Linköping, Jönköping and Kalmar. All included patients were identified using the digital software CSAM Cytodose (CSAM Health AS, Oslo, Norway), used for prescribing chemotherapy at all participating departments.

Inclusion criteria were patients with BTC or GBC ICD codes (C23.x, C24.x, and C22.1) that had received at least one dose of palliative first line GemOx at any of the participating centres between November 2011 and September 2020 and subsequently had received any type of second line systemic treatment.

Clinical data were collected from medical records with a structured case report form. All patients were followed until death or the 31st of December 2020, whatever came first.

As no targeted treatments (such as FGFR or IDH inhibitors) for aBTC had been introduced in routine care by September 2020, there were no molecular profiling or next generation sequencing data available and none of the participating patients had received any such treatment.

### Statistics

Statistical analyses were performed using SPSS Statistics v25 (IBM, Armonk, NY, USA) and R software version 4.5.1 (R Development Core Team, Vienna, Austria; http://www.r-project.org). Primary outcome was median overall survival (OS), counted from start of second line treatment until death or last follow up date. Secondary outcomes included time to treatment failure (TTF), counted from start of treatment until end of treatment, including discontinuation for any reason including toxicity as well as progression (clinical and/or radiological). TTF was chosen instead of progression free survival (PFS), based on the hypothesis that many patients would not have had time to undergo radiological evaluation before discontinuation of second line therapy. Median OS and TTF were estimated using Kaplan-Meier survival analysis and the significance of the difference between factors was calculated using Mantel-Cox log rank test. Hazard ratios were calculated using Cox regression analysis. p-values < 0.05 were considered significant. Variables in the univariable analysis with p-values < 0.2 were used in the multivariable analysis.

Variables with *p* < 0.05 in the multivariable analysis were used to establish an exploratory nomogram, allowing the estimation of 6- and 12-months survival probabilities as well as estimated median OS. The patients were then stratified into three different groups, with similar sample size, depending on total nomogram points. Patients with score < 137 was considered low risk, 137–173 intermediate risk and > 173 high risk. The cut-off values used for risk stratification were data-driven. Due to the exploratory nature of the nomogram, no internal validation or analyses to determine optimal cut-off points for the nomogram were made.

### Ethics

Ethical approval for this study was granted by the Regional Ethics Review Board in Linköping (diary number 2018/139-31). Due to the retrospective non-interventional design and the fact that the majority of patients were not expected to be alive at time of data collection, the Ethics Review board waived the requirement for informed consent.

## Results

In total, 121 patients received first line palliative treatment with GemOx at any of the participating centres during the study period. Fifty-seven (47%) of these received second line treatment of any kind and constituted the cohort for this study. The patients receiving second line therapy were younger (62 yeas vs. 67 years), had a better ECOG PS at start of first line treatment and to a lesser extent had locally advanced tumour at diagnosis. They also had a longer OS, counted from end of first line treatment (6.5 months vs. 2.0 months). (Table 1).

**Table 1:** Comparison between patients that did or did not receive second line treatment

	Patients receiving 2nd line treatment	Patients not receiving 2nd line treatment	*p*-value^a^
Age at primary diagnosis, median (range)	62 (22–74)	67 (45–82)	< 0.001^b^
ECOG at start of first line treatment, n (%)			
0	18 (32.7%)	17 (34.0%)	**0.021**
1	33 (60.0%)	20 (40.0%)	
2	4 (7.3%)	13 (26.0%)	
Locally advanced at primary diagnosis, n (%)			
Yes	8 (14.0%)	20 (37.0%)	**0.005**
No	49 (86.0%)	34 (63.0%)	
Distant metastasis at primary diagnosis, n (%)			
Yes	39 (68.4%)	28 (51.9%)	0.074
No	18 (31.6%)	26 (48.1%)	
OS from start of first line treatment (months, 95% CI)	11.1 (8.0-14.2)	6.8 (5.3–8.5)	**0.002** ^c^
OS from end of 1st line treatment (months, 95% CI)	6.5 (3.2–9.8)	2.0 (1.2–2.8)	**< 0.001** ^**c**^

^a^ χ2 test unless otherwise specified; ^b^Mann-Whitney U test; ^c^log-rank test

Sixteen patients (28.1%) had a history of previous curative intent surgery prior to commencing GemOx. The most common localizations (of the primary tumour) were pCCA (18 patients, 31.6%), followed by iCCA (16 patients, 28.1%), GBC (11 patients, 19.3%), and dCCA (8 patients, 14.0%). In further analyses, a simpler categorization into intrahepatic (iCCA) and extrahepatic (including pCCA, dCCA and GBC) BTC was used. The main reason for termination of first line treatment was disease progression (48, 84.2%) followed by toxicity (5, 8.8%). (Table 2).

Eight patients (14.0%) presented with Eastern Cooperative Oncology Group performance status (ECOG PS) 0 at start of second line treatment, twenty-seven (47.4%) with 1, and 14 (24.6%) with 2. The most used second line regimen was capecitabine monotherapy (37 patients, 64.9%) followed by fluorouracil (5-FU) with folinic acid in nine (15.8%) patients. Two patients received antibodies in addition to capecitabine with either bevacizumab or panitumumab. Eight patients received combination therapy with two chemotherapeutic drugs (generally a fluoropyrimidine combined with gemcitabine, irinotecan, or oxaliplatin). (Table 2).

The clinically used cut-off level for serum-albumin varies with age. In this study a cut-off of 36 g/L was used, corresponding to the lower limit of reference for patients < 70 years old, as 91% of included patients were in this age category.

**Table 2:** Patient, tumour and treatment characteristics

Patient, tumour and treatment characteristics	*n* (%)
Gender	
male	33 (57.9)
female	24 (42.1)
ECOG PS at start of 2nd line treatment	
0	8 (14.0)
1	27 (47.4)
2	14 (24.6)
Missing data	8 (14.0)
Previous curative intent surgery performed	
yes	16 (28.1)
no	41 (71.9)
Site of primary tumour	
perihilar	18 (31.6)
intrahepatic	16 (28.1)
gallbladder	11 (19.3)
distal	8 (14.0)
missing data	4 (7.0)
Plasma albumin at start of 2nd line treatment (g/L)	
≥36	17 (30.4)
<36	39 (69.6)
Reason for termination of 1st line treatment	
progression	48 (84.2)
toxicity	5 (8.8)
stable disease of remission	1 (1.8)
elevated liver enzymes	1 (1.8)
other	2 (3.5)
Regimen used for 2nd line treatment	
Capecitabine	37 (64.9)
5-FU	9 (15.8)
CAPIRI	3 (5.3)
FLIRI	2 (3.5)
GemCap	2 (3.5)
Tegafur/Oteracil/Gimeracil	1 (1.8)
CAPOX	1 (1.8)
Capecitabine + bevacizumab	1 (1.8)
Capecitabine + panitumumab	1 (1.8)
Age (years)	
≥65	23 (40.4)
< 65	34 (59.6)
Distant metastases at primary diagnosis	
yes	18 (31.6)
no	39 (68.4)
Locally advanced at primary diagnosis	
yes	8 (14.0)
no	49 (86.0)

### Survival

Median OS for the whole cohort was 4.1 months (95% CI 3.2-5.0) from start of second line treatment until death. The six- and twelve-month survival rates were 35.6% and 18.6%, respectively.

Cox regression univariable analysis was utilized to assess the impact of sex, previous curative intent surgery, plasma albumin at start of second line treatment, ECOG PS 0–1 vs. ≥ 2, site of primary tumour and PFS on first line treatment more than or equal to 6 months. ECOG PS 0–1 was associated with better OS (5.2 months compared to 2.9 months), with ECOG 2 having a hazard ratio (HR) of 4.7 (95% confidence interval (CI) 2.3–9.6, *p* < 0.001). First line PFS ≥ 6 months was similarly associated with better prognosis, with OS 5.8 months as compared to 3.3 in patients with PFS < 6 months under first line treatment (HR 2.1 95% CI 1.1–3.7, *p* = 0.016). A multivariable analysis was conducted, including variables with *p* < 0.2 in the univariable analysis (gender, baseline serum albumin, first line PFS, site of primary tumour, and ECOG PS). ECOG PS and PFS ≥ 6 months during first line treatment emerged as independent prognostic factors, as well as site of primary tumour. (Table 3).

**Table 3:** Median overall survival, from start of second line treatment, for different factors with univariable and multivariable analysis

Patient and tumour characteristics	OS (95% CI)	HR univariable (95% CI)	*p*-value	HR multivariable (95% CI)	*p*-value
Total cohort	4.1 (3.2-5.0)				
Gender					
male	4.4 (3.3–5.6)				
female	2.9 (1.7–4.2)	1.5 (0.9–2.6)	0.153	0.8 (0.4–1.6)	0.530
Curative intent surgery performed					
yes	2.9 (1.6–4.3)				
no	4.4 (3.4–5.4)	0.7 (0.4–1.3)	0.304		
Albumin (g/L)					
≥ 36	5.8 (2.2–9.5)				
< 36	3.6 (3.0-4.3)	1.6 (0.8–2.9)	0.154	1.8 (0.8–3.9)	0.154
ECOG					
0–1	5.2 (2.7–7.7)				
2	2.9 (0.2–5.5)	4.7 (2.3–9.6)	**< 0.001**	6.5 (2.6–16.5)	**< 0.001**
Primary site					
intrahepatic	4.0 (1.7–6.4)				
extrahepatic	3.7 (2.5–4.8)	1.7 (0.9–3.3)	0.115	2.8 (1.3–6.3)	**0.012**
PFS 1st line treatment ≥ 6 months					
yes	5.8 (2.3–9.4)	2.1 (1.1–3.7)	0.016	3.2 (1.5-7.0)	0.003
no	3.3 (2.4–4.3)				
Age					
≥65	4.2 (3.1-5.0)	1.0 (0.5–1.7)	0.865		
<65	4.0 (2.8–5.3)				

### Exploratory prognostic nomogram

**Fig. 1:**
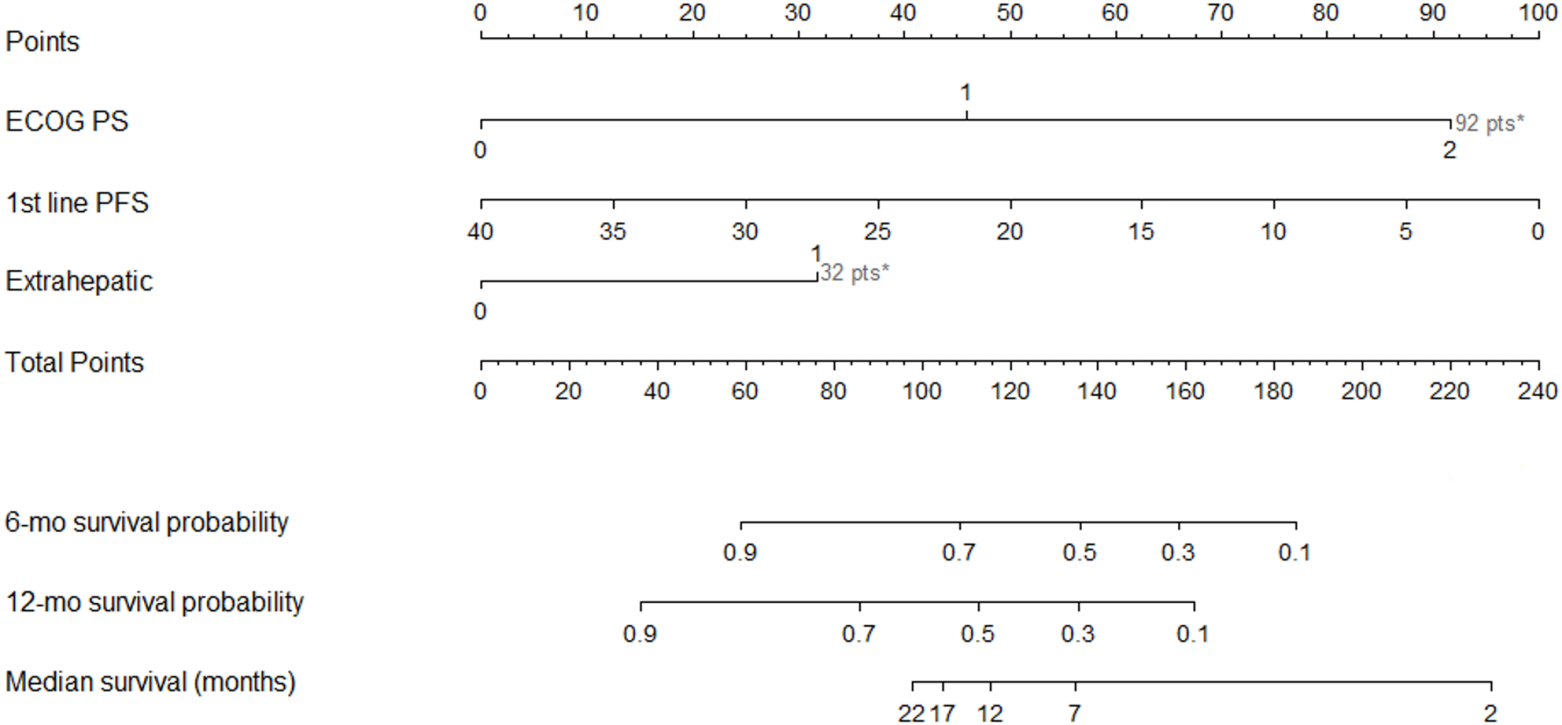
Prognostic nomogram for prediction of overall survival probability at start of 2nd line chemotherapy

A nomogram was established, incorporating the factors proven associated with worse overall survival in multivariable analysis, i.e. ECOG PS > 1, PFS < 6 months during first line treatment and extra-/intrahepatic disease (Fig. 1).

**Fig. 2:**
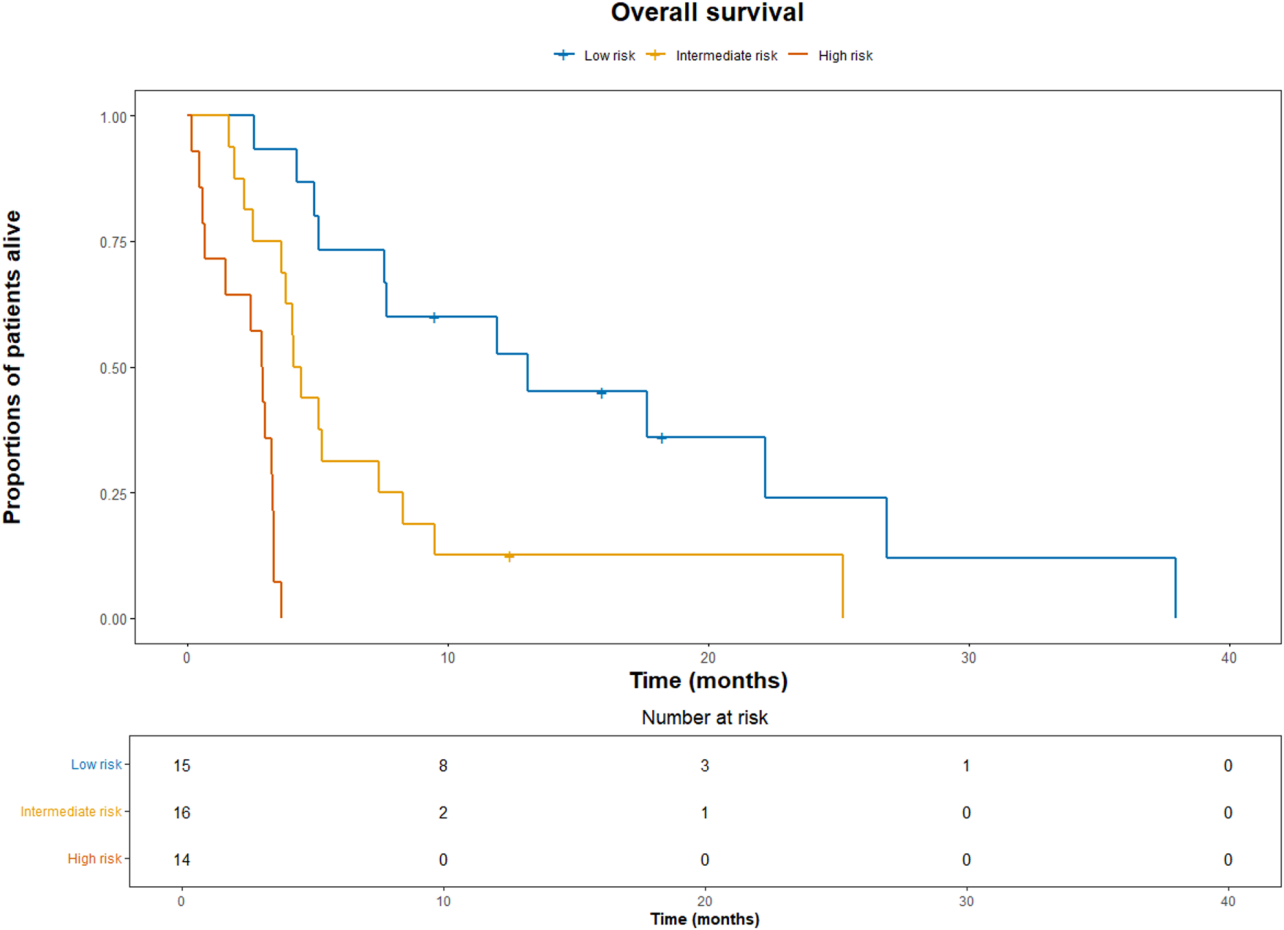
Kaplan-Meier diagram showing overall survival for patients in low, intermediate and high-risk groups. The number of events was 12 in the low-risk group, 15 in the intermediate-risk group, and 14 in the high-risk group

A prognostic model, depending on total points from the nomogram, was explored. Patients were divided into three groups. Total points < 137 were considered low risk (15 patients, OS 13.1 months), 137–173 intermediate risk (16 patients, OS 4.3 months), and > 173 high risk (14 patients, OS 2.9 months). (Table 4; Fig. 2).

**Table 4:** Median overall survival from start of second line treatment and univariable analysis for patients in low, intermediate and high-risk groups

Risk (*n*)	OS (95% CI)	HR (95% CI)	*p*-value
Low (15)	13.1 (7.6-NA)	1	-
Intermediate (16)	4.3 (3.6–9.5)	2.9 (1.3–6.7)	**0.01**
High (14)	2.9 (1.5–3.4)	14.4 (7.2–83.1)	**< 0.001**

### Time to Treatment Failure (TTF)

Median TTF for the whole cohort was 1.8 months (95% CI 1.5–2.2). (Table 5).

The same factors used in the overall survival analysis were evaluated in a Cox regression model for TTF, with ECOG PS being the only parameter reaching statistical significance. Patients with ECOG 0–1 had longer TTF compared to ECOG 2 patients (2.5 months compared to 0.7 months, HR 3.4 (95% CI 1.7-7.0). A multivariable analysis was conducted, with only ECOG PS reaching statistical significance (Table 5).

**Table 5:** Time to treatment failure for different factors with univariable analysis

Patient and tumour characteristics	TTF (95% CI)	HR univariate (95% CI)	*p*-value	HR multivariable (95% CI)	*p*-value
Total cohort	1.8 (1.5–2.2)				
Gender					
male	1.8 (1.2–2.5)				
female	1.5 (0.7–2.3)	1.1 (0.6–1.9)	0.764		
Curative intent surgery performed					
yes	1.6 (1.2-2.0)				
no	1.9 (1.3–2.5)	0.7 (0.4–1.2)	0.196	0.7 (0.3–1.3)	0.247
Albumin (g/L)					
≥ 36	2.1 (0.1–4.1)				
< 36	1.8 (1.3–2.3)	1.4 (0.8–2.6)	0.224		
ECOG PS					
0–1	2.5 (0.7–4.4)				
2	0.7 (0.0-1.5)	3.4 (1.7-7.0)	**< 0.001**	2.8 (1.3–5.8)	**0.006**
Primary site					
Intrahepatic	1.9 (1.0-2.8)				
Extrahepatic	1.6 (1.4–1.9)	1.4 (0.8–2.5)	0.297		
PFS first line treatment ≥ 6 months					
yes	3.1 (1.2–5.1)				
no	1.6 (1.3–1.9)	1.5 (0.9–2.6)	0.159	1.6 (0.9–3.6)	0.136
Age (years)					
≥ 65	2.1 (1.0-3.2)	0.7 (0.4–1.2)	0.181	0.7 (3.7–1.2)	0.136
< 65	1.6 (1.3-2-0)				

### Palliative care admission and treatment in end of life

Thirty-seven patients (64.9%) received specialized palliative care. Ten (17.5%) were already admitted to a specialized palliative care team prior to start of second line treatment. Median number of days from start of second line treatment to inclusion was 61 (range 0-789).

Sixteen patients (28.1%) received their last dose of chemotherapy within their last 30 days of life. Median duration from end of second line chemotherapy to death was 55.5 days (range 0-765).

## Discussion

Biliary tract carcinomas are a rare group of cancers that contribute to significant morbidity and high mortality rates for those affected. Novel treatment options, including combined chemo-immunotherapy in first line and targeted treatments in the second line, have emerged as options in selected cases, but for a considerable subset of patients conventional chemotherapy remains the only viable option to control the disease. Selective FGFR inhibitors have shown effect on PFS in patients harbouring FGFR-2 fusion (10–15% of patients with iCC) [11]. In patients with IDH-1 mutant tumours (13% of patients with iCC, 1% of patients with extrahepatic cholangiocarcinomas), IDH-1 inhibitors have shown an improvement in PFS [13]. Other possible targets such as HER-2 amplifications, BRAF V600E mutations and NTRK fusions have also shown promising results [14–16]. Gene alterations vary depending on anatomical location, highlighting the heterogeneity of BTC and periampullary carcinoma [17, 18]. However, the vast majority of patients will not be eligible for or have access to such treatments, and conventional chemotherapy remains the mainstay in these cases.

This study presents real-world data on patients who received second line treatment after progression on first line palliative GemOx.

Our results show a very poor prognosis in general, with median OS of 4.1 months and median TTF of only 1.8 months. This is somewhat worse than what was observed in previous studies, where OS ranged from 3.5 to 10 months, and PFS ranged from 1.4 to 6.5 months [19]. However, a similar study by Lowery et al. [20] reported a TTF of 2.2 months, although a better OS of 11 months. Brieau et al. [21] reported a OS of 6.7 months and median PFS of 3.2 months.

It is also noteworthy that the most commonly used second line regimen in our study was monotherapy with capecitabine, which is a standard treatment for BTC – particularly in the adjuvant situation [22] - but may be less effective compared to more complex drug combinations in more advanced stages of the disease. Several prior studies have suggested a potential advantage of combination therapies [23, 24], yet these regimens might not always be possible to use in this patient group, e.g. due to poor PS or remaining side effects from previous treatments. Due to the small sample size and presumed selection bias (e.g. fit patients more likely to receive combination regimens etc.), statistical comparisons between different second line therapies were not deemed feasible or appropriate in this cohort.

As expected, low PS was associated with worse OS, a finding that has been confirmed in multiple previous studies [25, 26]. An important observation in our study was that patients with progression-free survival (PFS) of ≥ 6 months during first line treatment had a significantly better prognosis, which is consistent with previous findings showing that longer PFS after first line therapy is a predictor of better treatment outcome and long-term survival following second line attempts [27]. Based on these factors, we explored a prognostic model with three subgroups of patients with different survival outcomes. Our OS curves were comparable with the prognostic model by Fornaro et al. [27], but with the benefit of using a nomogram for risk stratification, i.e. using continuous rather than categorical variables. Notably, the nomogram was developed using a small sample size and without validation, therefore it should be interpreted as hypothesis-generating only. With a OS of only 2.9 months in the high-risk group, approaching the OS of non-2nd -line treated patients, it is reasonable to question whether patients with a higher PS, extrahepatic disease and short time to failure on first line treatment will benefit from second line treatment, unless the patient is eligible for novel targeted treatments. On the other hand, in patients with good PS, intrahepatic disease, and a history of long-lasting response to first line therapy, it is reasonable to consider second line chemotherapy.

To date there are few studies investigating second line treatment after first line treatment with GemOx. Present studies have focused on treatment beyond progression following failure on first line GemCis (rather than GemOx), do not specify the platinum component being used, or do not have a real-word approach. There is however a similar French study by Brieau et al., investigating second line therapy after failure of gemcitabine-platinum combination, where most patients did receive GemOx. The most commonly used second line treatments in their study was FOLFIRI/XELIRI followed by LV5FU2 + cisplatin, 5-FU/capecitabine, FOLFOX/XELOX and Sunitinib. The patients in this French study were more often treated with combination regimens, which might explain the slightly better OS of 6.7 months [21]. It is possible that the patients in this trial were fitter than the subjects in our cohort, naturally having better prognosis and also more likely being eligible for multi-drug regimens.

The ABC-06 study [12] investigated FOLFOX plus active symptom control (ASC) vs. ASC alone, and found that the group receiving FOLFOX had a higher OS of 6.2 months compared with 5.3 months in the group receiving only ASC. This was higher than the OS observed in our study in both groups, which might be explained by the fact that only patients with ECOG PS 0–1 was included in the ABC-06 study.

The first line regimen used in the ABC-06 study was GemCis, which has a different toxicity profile, with more haematological toxicity, compared with GemOx, which is associated with more neurotoxicity [28]. It is possible that residual neurotoxicity influenced the ability to tolerate multidrug regimens, which might explain the low number of patients receiving these regimens in our study.

We found that 28.1% of patients received chemotherapy in the last 30 days of life, this is slightly lower than presented in previous research, ranging from 32 to 52% of the patients with disseminated cancer receiving treatment in the last month of life [29, 30]. It is however higher than what we found in those patients who received only one line of treatment, where only 18.5% of patients received treatment in the last 30 days of life [9].

Although this study provides valuable insights into treatment patterns and outcomes of second line treatment in aBTC, it has some limitations. First, it has a retrospective approach meaning that confounding factors and selection bias in terms of treatment choice are not negligible. Second, there is no control group, which prevents a comparison of the results from our cohort with a group that did not receive treatment, and no causal inference can be made. This weakness is inherent to all studies of this type; naturally, patients were selected to either second line chemotherapy or best supportive care based on their fitness, and it would not be ethically feasible to exclude patients who were considered fit from treatment or vice versa, i.e. offering chemotherapy to patients who were considered too weak to tolerate such treatment. We did, however, compare patients who did not receive second line treatment with those who did. Patients receiving second line treatment were younger, had a better PS at the start of first-line treatment, were less likely to have a locally advanced tumour at diagnosis, and had a longer OS. In our cohort, another selection bias is evident, as the majority of patients are younger than 65 years, whereas the peak incidence age for BTC is approximately 75 years. This is however not surprising, as it is plausible to believe that elderly patients with progressive BTC are less likely to be fit for any further cytotoxic therapy than younger ones. Another limitation is the relatively small sample size, though the number of patients included is noteworthy given the rarity of BTC. Also, the design of our study meant that all eligible patients in a region with three cancer centres and an accrual period of eight years were included. The Swedish cancer care is publicly funded and there are no private or alternative oncology care givers in this geographical region, meaning that the studied population was a true real-world cohort.

This study confirms the poor prognosis of patients with progression after first-line GemOx treatment for aBTC, and we identified several prognostic factors that are important for predicting patient survival and treatment response. A substantial proportion of the patients with aBTC who have received GemOx as first-line treatment had a very poor outcome, and the short OS and TTF observed indicate a dire need for new, more effective treatment options. The study also emphasizes the importance of developing individualized treatment strategies based on biomarkers to improve treatment outcomes.

Prospective randomized clinical trials comparing alternative second line treatment regimens represent the gold standard for generating robust evidence on efficacy and safety, although they are often challenging to design and conduct. Research into biomarkers that can predict treatment response and survival would be an important step toward tailoring treatments and improving patient outcomes. The rapid advancements in genomics and precision medicine also open the door to testing how genetic alterations and mutations impact responses to different therapies.

## Conclusion

This study highlights the poor prognosis of patients undergoing second line treatment following first line GemOx for aBTC. Sustaining response on first line treatment, good ECOG PS and intrahepatic disease indicate slightly better prognosis. There is a dire need for novel treatment options for patients refractory to first line treatment in aBTC.

## Data Availability

The datasets used and/or analysed during the current study are available from the corresponding author on reasonable request.

## Abbreviations

GemOx: Gemcitabin Oxaliplatin
BTC: Biliary tract cancer
aBTC: Advanced Biliary tract cancer
iCCA: Intrahepatic cholangiocarcinoma
pCCA: Perihilar cholangiocarcinoma
dCCA: Distal cholangiocarcinoma
GBC: Gallbladder cancer
GemCis: Gemcitabine Cisplatin
FOLFOX: 5-fluorouracil, folinic acid, and oxaliplatin
OS: Overall survival
TTF: Time to treatment failure
PFS: Progression free survival
HR: Hazard ratio
CI: Confidence interval
ECOG PS: Eastern Cooperative Oncology Group performance status
FOLFIRI/XELIRI: leucovorin calcium (folinic acid), fluorouracil, and irinotecan hydrochloride
LV5FU2: Leucovorin and bolus and infusional fluorouracil
5-FU: Fluorouracil
FOLFOX/XELOX: Folinic acid, fluorouracil and oxaliplatin
ASC: Active symptom control
